# Epidemic Cholera

**Published:** 1854-02

**Authors:** 


*Epidemic Cholera.*—Hon. Hamilton Fish, Senator from New York—the same gentleman who, many of our readers may remember, so hospitably entertained the members of the American Medical Association, during its session in that city in May last—on the first day of the session of the present Congress, before that body was fully organized, and before the reception of the President's Message, and other public documents, offered the following resolution, which was afterwards adopted:

*Resolved*, That a select committee of five be appointed to consider the causes, and the extent of the sickness and mortality prevailing on board emigrant ships, on the voyage to this country, and whether any, and what further legislation is needed for the better protection of the health and lives of passengers on board such vessels.

Our readers may remember that in the Reporter for September 30th, in announcing the prevalence of cholera in its epidemic form, in some parts of Europe, we expressed the fear that it would, ere long, invade our shores, as it seemed to be pursuing much the same course it did in the memorable epidemics of 1832 and 1849. In England the scourge seems to have met with a temporary check, yet it still lingers in the purlieus of Cripplegate and St. Martin's-in-the-Fields, in London, and in other of the faubourgs of that, and other of the maritime cities of Europe. It would be well for us, if it would remain there; but, while such a tide of emigration, which for the past few months, has been at the flood, is pouring its thousands on our shores, from these very haunts of disease and pestilence, it seems hardly possible for us to escape. Nor are we likely to do so, in view of the terrible mortality on emigrant ships during the month of November, referred to in the resolution of Mr. Fish, quoted at the head of this article. Of the whole number of emigrant vessels that arrived at the port of New York alone, twenty-eight had cholera on board. Of 13,762 emigrants who took passage on those twenty-eight ships, no less than 1,141 died of cholera, while from four to five thousand were afflicted with it during the passage. The epidemic generally broke out when the vessels were two or three days out, and ceased when they reached soundings on this side.

We have no evidence other than newspaper accounts, that this plague is the true Asiatic cholera, but, if it is not, it is at least blazing a pathway, which may the coming year, be a highway for that pestilence to invade our shores. This, it certainly will be, unless the present awful mortality is arrested. That this condition of things on board our emigrant ships is not without local causes, may be gathered from statements in the newspaper press, describing the condition of things on board those vessels. A writer in the *New York Daily Times*, of Dec. 1, gives the following description of the revolting state of affairs on board one ship, from which out of 700 passengers, 90 lives were lost on the passage—

"The place where these miserable 700 were suffering, was so dark that nothing could be seen without a light; the emigrants would not tell of a death, and in some instances, three or four would continue in a berth for two or three days beside a corpse, and the discovery was only made at last, by the nose of the sexton.

"The filth in this lower region was nearly knee deep, and to go through it with the screams and groans of the suffering, added to the offensive filth, gave you (as a minister on board remarked,) a distinct idea of hell.

"The filthiness of these emigrants, and their destitute condition, were described, but their recital would be too gross to repeat. It should however be made the subject of careful investigation, by the proper authorities. Talk of the horrors of a slave ship, when such horrors are at our very doors!"

It is a matter of sincere congratulation that Senator Fish, in bringing this important subject to the attention of Congress, has shown his determination to use his influence in legislating for the *health*, as well as the *pockets* of his constituency, and if there is any truth in the proverb, "Health makes wealth," by promoting the one he will surely advance the other.

We believe that but few cases of this sea plague, be it cholera or no, have as yet occurred in any of our Atlantic cities, though large numbers have been sent to the Quarantine Hospitals; and it is to be hoped that such preventive measures will be adopted by our Boards of Health, as will effectually close the avenues to its appearance among us.

New Orleans has not been so fortunate; for it appears that that ill-starred city, which is just recovering from the effects of the recent severe epidemic of yellow fever, is now threatened with the horrors of cholera epidemic. We are not, however, without hope that the cool weather of the winter months, aided by the adoption of proper sanatary regulations, may have a tendency to avert the threatened danger.—*New Jersey Medical Reporter.*

